# A new approach to cold surge classification in East Asia

**DOI:** 10.1038/s41598-021-02873-0

**Published:** 2021-12-08

**Authors:** Anupam Kumar

**Affiliations:** 1 Interdisciplinary Graduate School, Institute of Catastrophe Risk Management, Nanyang Technological University, Singapore, Singapore; 2 Centre for Climate Research Singapore, Meteorological Service Singapore, National Environment Agency, Singapore, Singapore

**Keywords:** Climate sciences, Atmospheric science, Atmospheric dynamics

## Abstract

Evidence showing a strengthening of intense cold surge event (CSE) in East Asia, e.g. CSE of Jan 2016 and Jan–Feb 2008, is focusing attention towards the science of CSE onset prediction. Predicting the onset of such strong CSEs remains elusive as the extent of these surges varies over spatial and temporal scales. Changes in radiative cooling over Siberia in winter as potentially affected by changes in the Arctic are further expected to influence CSE occurrences in East Asia. Moreover, unprecedented and long lasting CSEs in East Asia have a very distinct Jet Stream pattern via their shifts from the climatological mean, influencing the lower troposphere. Here, using modelling framework we propose a new relationship between Jet Stream and Aleutian Low for identifying and characterizing atmospheric process that leads to CSEs in East Asia. Our results reveal new insight into the mechanisms of CSEs occurrences, the absence of which may lead to major constraints on reducing CSE onset prediction error.

## Introduction

The outbreak of cold air masses over Siberia, commonly referred to as Cold Surges is one of the most distinct extreme weather events in East Asia during the north east monsoon^1^. Strong CSE contributes greatly to snowfall and freezing precipitation and can lead to disasters in East Asia^2^. Moreover, their interaction with the tropical warm water of South China Sea is considered as the root causes of severe weather situations in South China and nearby regions^3,4^. Here we show that the onset of CSE besides being strongly influenced by Siberian High (SH), is also driven by the progression of Jet Stream (JS) in upper air that significantly influences Aleutian Low (AL) in the lower troposphere. These interactions not only contribute to single CSE onset but also explain the multiple episodes of surges within a surge duration (e.g. over Jan 2011, Jan–Feb 2008, and Dec 2005).

CSE onset imposes three major effects on convective patterns at the synoptic scale in East Asia. The first contributes towards deep convection over the maritime continent marked by a general increase of convective activity^5^ leading to heavy rainfall and can result in severe flooding in the equatorial zone^6^. Secondly, since CSE progression is marked by sudden changes in surface pressure and temperature^7^, its onset can have severe impacts both on human health, leading to sudden death^8^ and affecting the environment through air pollution^9^. Lastly, long lasting CSE (i.e. duration) can be devastating (e.g. CSE in Jan–Feb 2008), and such CSEs have also been linked to the genesis of another meteorological hazard, that of tropical cyclones^10^.

The Fifth Assessment Report (AR5) of the Intergovernmental Panel on Climate Change (IPCC) shows substantial evidence that global climate has undergone significant warming, though the climate change effect at regional scales on CSEs occurrences is still being debated^11,12^. This is because nearly every extreme event including CSE occurrence can be strongly linked firstly to temperature with high confidence because of its direct connection with the surface temperature. Conversely, we have much less confidence in linking such extreme event occurrences to atmospheric circulations via its interaction with the lower troposphere affecting the local climate. These different views of the thermodynamic and dynamic responses of CSEs onset have led to different criteria adopted in the East Asia regions (e.g. Korea, Japan, south China, Taiwan etc.) for declaring region specific CSE onset.

SH amplification is a well-accepted necessary factor for the genesis and growth of CSEs^13^. It is also well known that the progression of JS over East Asia has a significant influence over weather and climate systems in the Asian-Pacific region as evidenced from its role in monsoon and storm track activity, cyclogenesis, frontogenesis, and blocking^14,15^, whereas the AL plays a key role for the North Pacific climate^16^. Recent research demonstrates detectible evidence of JS connectivity to an unprecedented cold surge over Jan–Feb 2008^17^, which was considered as a major forecasting failure^2^. We hypothesize that inadequate representation of these processes is responsible for non-robustness in representing circulation response to CSE onset. Here we consider both the progression of JS and AL, specifically the strengthening of a jet streak embedded within the JS at upper atmosphere and the resulting intensification of AL at the lower troposphere, respectively as a linkage for an improved understanding of CSE onset.

## Synoptic pattern and nature of CSE onset

**Figure 1:**
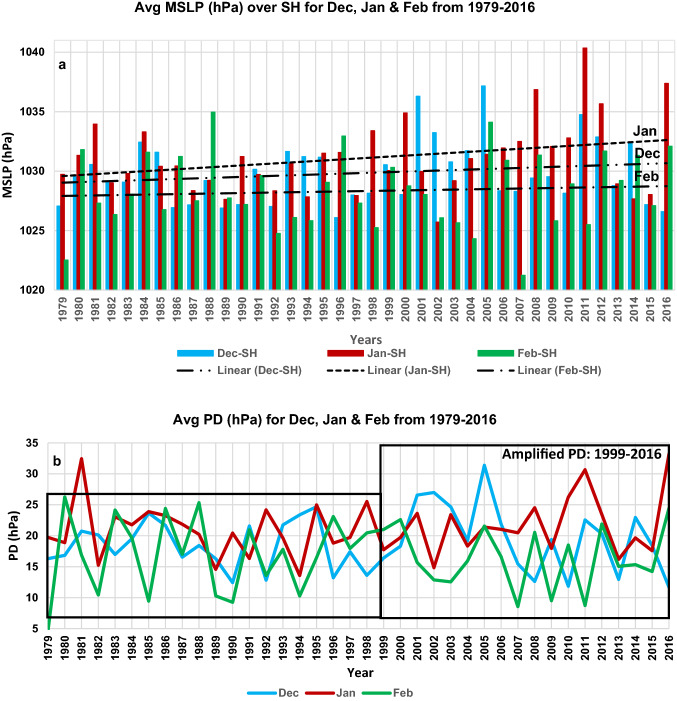
Dominance of SH and PD during North East Monsoon periods. (**a**) Average MSLP (hPa) over the SH domain and (**b**) Average PD (hPa) for Dec (blue), Jan (red) and Feb (green) from 1979–2016. Dotted lines in (**a**) indicate a linear trend for Dec, Jan, and Feb showing a noticeable trend for Jan with a p-value of 0.02619 from Mann–Kendall test. (Data source: European Centre for Medium Range Weather Forecast (ECMWF), ERA-Interim).

On a synoptic scale, cold surges are strongly affected by aspects of atmospheric circulation such as JS, Monsoonal system, and surge tracks^18–20^ whereas on a seasonal scale their physical links to the atmospheric circulation play a key role in predicting their anomalous patterns^21^. How dominant a surge would result strongly depends on the prevailing synoptic situation at that time. For example, it is well-known that SH amplification during the North East Monsoon (Dec to Feb) leads to outbreaks of cold air masses over Siberia. Figure 1a shows the monthly mean sea level pressure (MSLP) spatially averaged over the SH domain (defined here as over 40° N–60° N and 80° E–120° E) from 1979–2016 for Dec, Jan, and Feb. The highest MSLP of 1040 hPa is attained in Jan (2011) followed by 1037 hPa in Dec (2005) and 1034 hPa in Feb (1988). During this 38-year period, there is an overall increase in the MSLP strength for Jan whereas no significant trend is observed for Dec and Feb. This observation is consistent with the recent research that shows Jan is the most critical month for the outbreak of cold air mass^17^ and that the SH has been intensifying gradually^22^. This strengthening is further attributed as a response to Arctic amplification as related to albedo changes from loss of snow and sea ice, and the presence of heat-trapping clouds and water vapor^23^. Figure 1a further illustrates the SH intensification is well marked during the intense cold air outbreak as evident during CSEs of Dec 2005, Jan 2011, Feb 1988. However, during the onset of unusual CSEs that occurred during Jan 2016, Jan–Feb 2008, Dec 2009 the SH intensification was found to be comparatively lower. Recent research shows that the intensity of atmospheric circulation over SH to the surface environment over the AL, as characterized by a pressure difference (PD) between the two leads to a more robust indicator of cold air progression in East Asia^17^. Here the AL domain is defined as 25° N–75° N and 160° E–130° W for determining the monthly average AL MSLP used in the computation of the PD. Our analysis shows that the amplified period of PD is more evident for the latter period of 1999–2016 (Fig. 1b) as compared to the earlier period from 1979 to 1998, with exception of Jan 1981, which was characterized by a deep polar vortex and a strong planetary wave^24^. This suggests that there is a recent increase in PD amplification. Thus e.g. Jan 2016 had the strongest PD of 33 hPa in the last 38 years followed by Jan 1981. For Dec, the highest PD of 31 hPa was observed in 2005, while for Feb the highest PD of 26 hPa was observed in 1980. This further shows that Jan is the most crucial month for the progression of cold air outbreak followed by Dec and Feb. Moreover, this amplifying period is also coincident with the warmer decadal period, 2001–2010 which was the warmest on record, with five recent years, viz., 2016 (1.2 °C above preindustrial baseline), 2015, 2017, 2019 (all three years are 1.1 °C above pre-industrial baseline), and 2018 (1.0 °C above pre-industrial baseline) being the warmest as reported by World Meteorological Organization^25,26^. However, regional variation of PD could be a complex process, resulting in varying intensities of CSEs at different locations due to changes experienced in the atmosphere–ocean circulation systems. Thus, the ability to accurately predict the progression of CSEs necessarily needs an understanding of the dynamical processes that significantly influence the synoptic patterns.

## Connection between JS and AL

**Figure 2:**
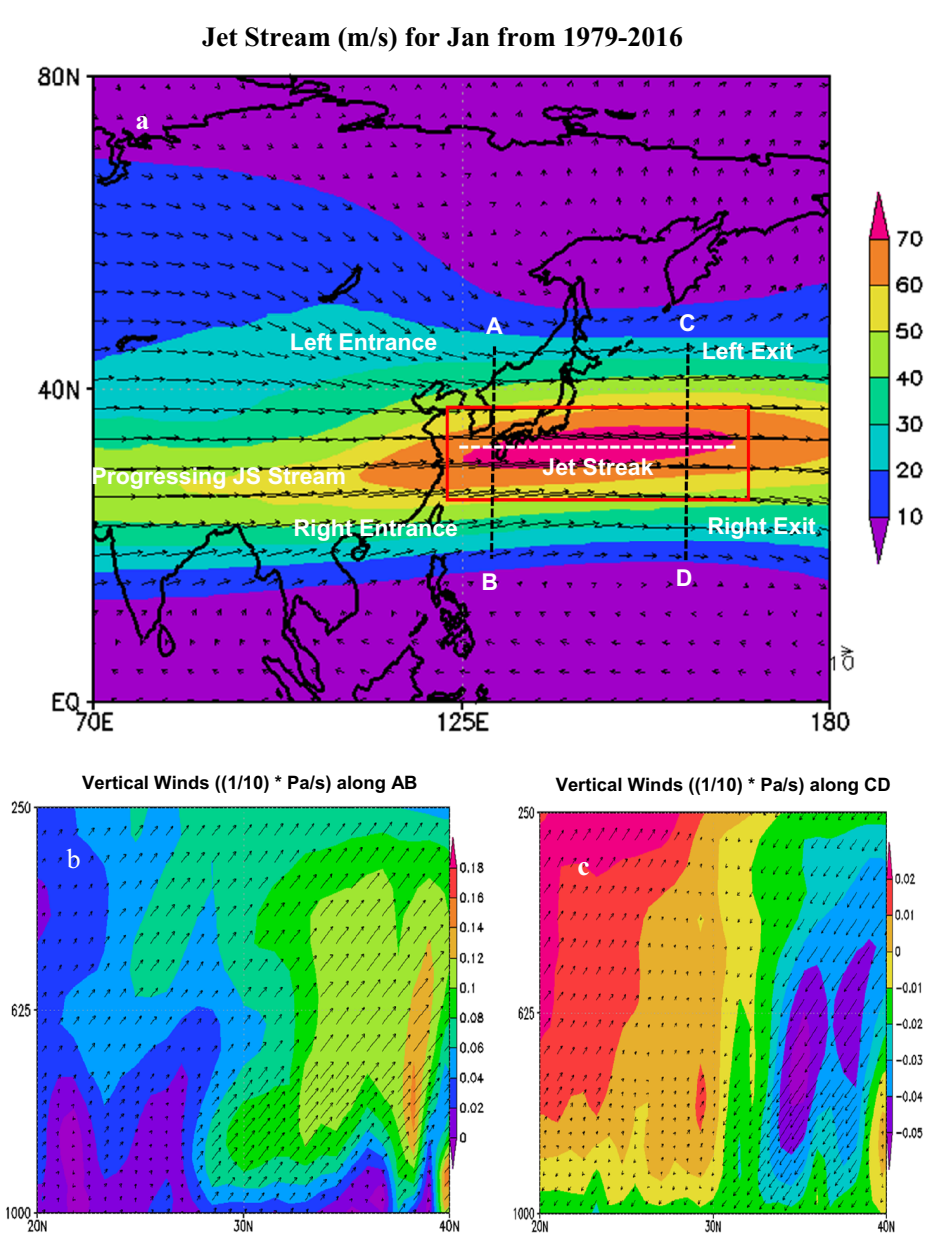
Contrast between converging and diverging winds at JS entrance and exit. Monthly average for Jan over 1979–2016 (**a**) JS speed (m/s) at 250 hPa, (**b**) cross section of JS along AB, (**b**) cross section of JS along CD.

**Figure 3:**
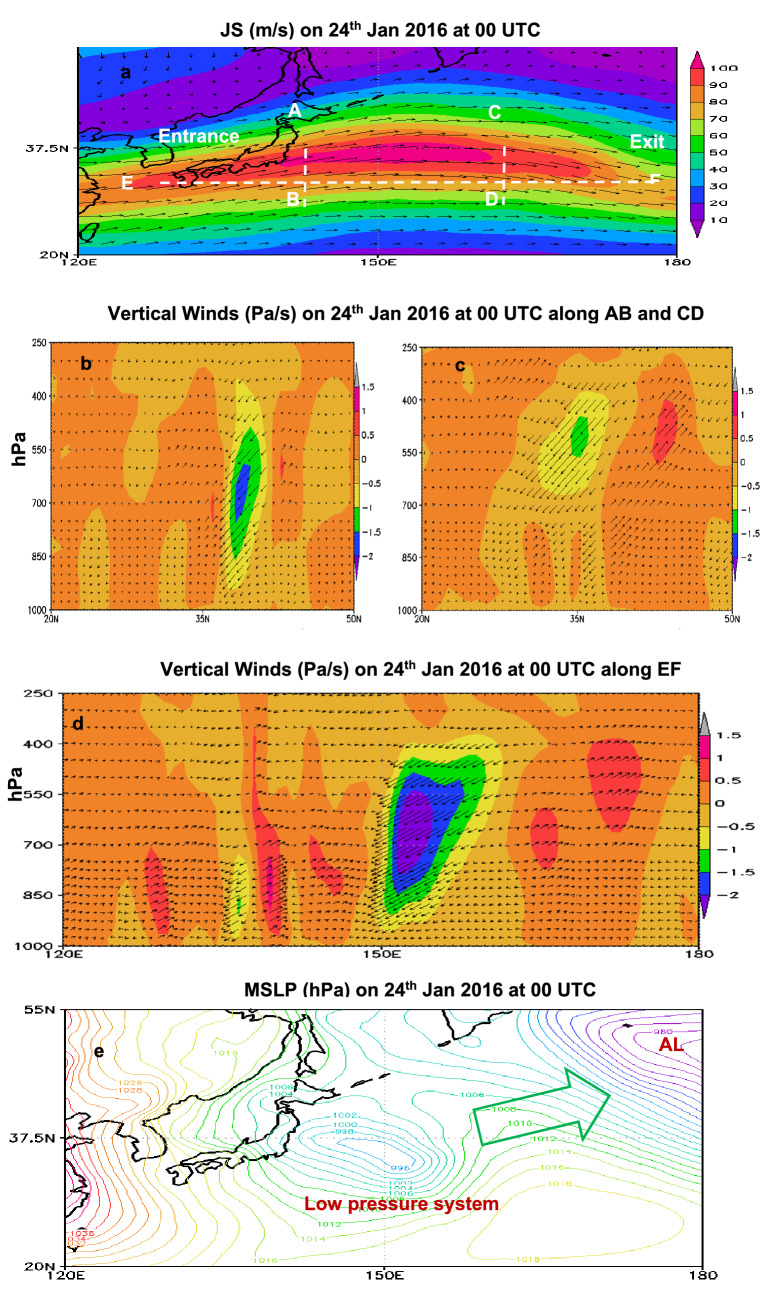
Diverging winds on 24th Jan 2016 from JS exit leading to the formation of a low pressure system at the surface. (**a**) JS (m/s) at 250 hPa, vertical cross section of JS (**b**) along AB at 140° N (**c**) along CD at 160° N, (**d**) along EF at 36° N, and (**e**) formation of low pressure system at the surface. Green arrow in (**c**) represents the eastward progression of low pressure system towards AL.

The physical process of the upper troposphere needs to be considered as they are likely related when explaining the mechanisms for cold air outbreak and CSE progression. Our analysis further shows that for the month of Jan, a jet streak within JS defined as extending from 125° E to 165° E (Fig. 2a, pinkish color) reaches a monthly-averaged maximum speed of 70 m/s at its center. As the air masses enter the JS it accelerates into the upstream side of the streak which we term here as an ‘entrance’. The accelerating air masses reach their maximum speed at the core of the JS i.e. center of the jet streak and start decelerating which we term here as an ‘exit’. These accelerating air masses as it enters jet streak shows two entrance regions around the jet streak, referred to here as the left entrance and right entrance regions, as seen in the vertical cross section along with AB. There is convergence at the left entrance and divergence at the right entrance (Fig. 2b). As the air masses exit jet streak, they decelerate and create divergence at the left exit and convergence at the right exit (Fig. 2c), as shown in the cross section along CD. These regions indicate warm air rising in the right entrance region and cold air sinking in the left entrance region (Fig. 2b). Whilst cold air is sinking in the right exit region, and warm air rising in the left exit region (Fig. 2c). Such changes in the upper air thus significantly influence the pressure systems at the lower troposphere. For example, during the CSE of Jan 2016, the jet streak attained a maximum speed of 100 m/s which is well above its climatological Jan mean of 70 m/s as the JS progressed over the western Pacific (Fig. 3a). The cross sectional plots along AB and CD show the rising and sinking air masses (Fig. 3b,c) as resulting from the progression of the diverging air masses at the entrance and exit inducing low level convergence, acceleration of lower winds, and enhanced rising motion towards the upper level (Fig. 3d). This further resulted in the lowering of the surface pressure beneath the atmospheric column, leading to the formation of a low pressure system (Fig. 3e). Once formed the low pressure system progresses eastward in the direction as inherited from the diverging air masses of the jet streak i.e. in the JS progression direction. It later merges with AL, and consequently intensifying the AL system (Fig. 3c). Therefore, the intensification of jet streak contributes to the genesis of the low pressure system which significantly influences the progression of CSEs in terms of their intensity. The evolution of the synoptic weather experienced during the surge month allows an overview of the synoptic scale pressure systems that trigger CSE conditions. The synoptic composites of the 31 days average of strengthening of jet streak wind speed (where the jet streak anomaly is 6.67 m/s above the 1981–2010 average) and intensification of AL (where AL MSLP anomaly is (−) 6.14 hPa below 1981–2010 average) leading to the onset of CSE 2016 are shown in supplementary Fig. 1. The cross sectional variations in jet streak wind speed and MSLP presented in the figure show the synoptic structure associated with the CSE that leads to its intensifying and weakening stages (represented in Fig. 5 on the mechanism). The cross sections, analyzed from the reanalysis dataset, are centered at 35° N for the jet streak and 36° N for MSLP based on the climatological mean with the longitudinal extent of 29 and 17° respectively. The air as it enters the jet streak (extreme left) starts accelerating and reaches the maximum speed and as the air leaves the jet streak it decelerates (extreme right). During this period within the same longitudinal domain (marked within the red box) sudden intensification of AL MSLP is observed. This shows that the influence of the progression of easterly jet stream brings unstable conditions over the AL domain, as observed by the sudden decrease in AL MSLP in contrast to the gradual increase in AL MSLP for the domain that is not under the direct influence of jet streak wind speed.

**Figure 4:**
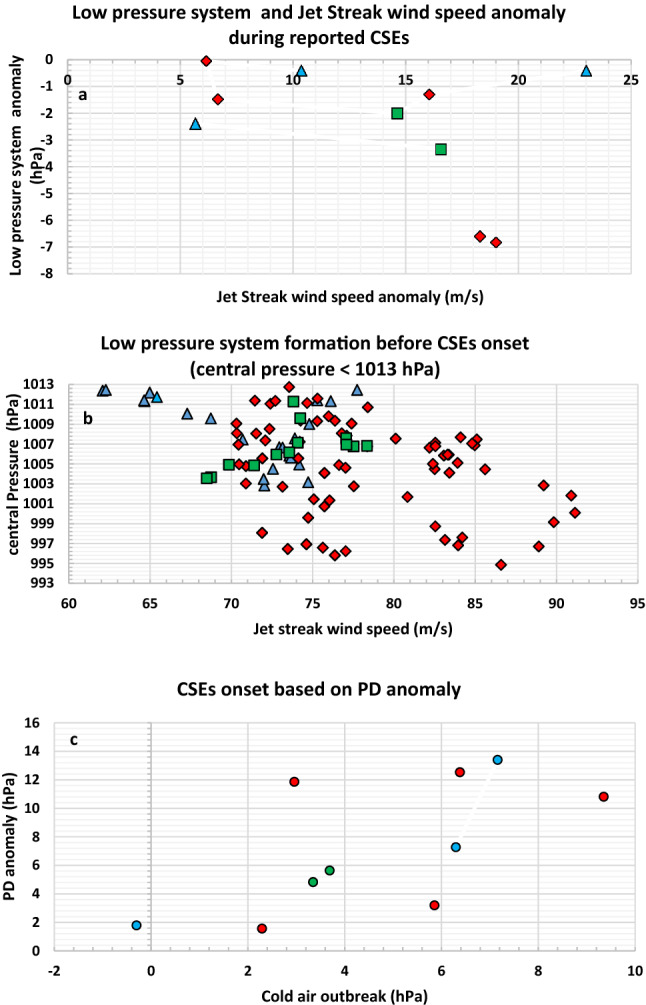
Connection between low pressure system and intensity of jet streak wind speed during reported CSEs. Scatter plot of jet streak wind speed anomaly verses low pressure system anomaly relative to 1981–2010 climate normal period during reported CSEs (**a**). Formation of low pressure system based on the intensity of jet streak wind speed for the months of Dec (blue-24 data points), Jan (red-84 data points), and Feb (green-13 data points) during reported CSEs period at 00, 06, 12, and 18 UTC (**b**). CSE onset based on PD anomaly versus Cold air outbreak anomaly relative to climate normal.

**Figure 5:**
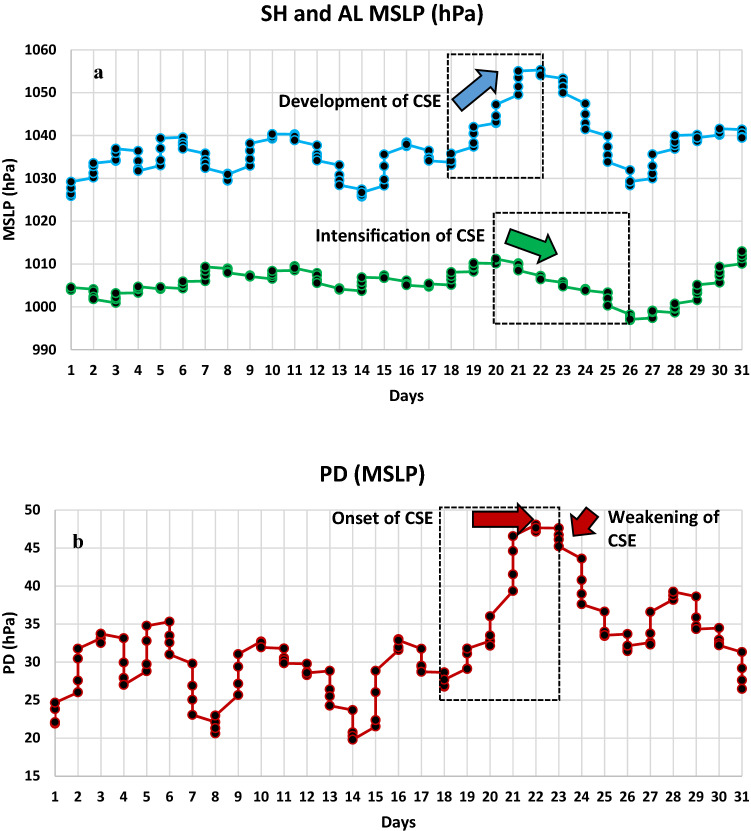
Schematic diagram of CSE onset procedures. (**a**) Sudden increase in SH MSLP leads to the outbreak of cold air masses and the corresponding drop in AL MSLP values intensifies the surge; (**b**) A well-established PD marks the onset of CSEs, whereas a dropping PD weakens CSEs progression.

As noted above, we analyzed the formation of low pressure systems due to the progression of the jet stream during the reported CSEs period (please see Table 1 for a list of reported CSEs). For all these events, jet streak wind speed has a positive anomaly while low pressure system has negative anomaly relative to the climate normal (Fig. 4a). This shows the intensification of low pressure systems with the strengthening of jet streak wind speed. Moreover, we also observed the formation of series of low pressure systems before the onset of the CSEs. These low pressure systems are seen to be most intense and more frequent during the month of Jan, followed by Dec, and Feb depending on the strength of the jet streak wind speed (Fig. 4b). This further confirms that jet stream progression results in the formation of low pressure systems which progress eastward following the direction of the jet stream to intensify AL. Intense AL well establishes PD leading to the progression of cold air masses at lower latitudes as CSEs (Fig. 4c). Therefore, with the outbreak of cold air mass and under the influence of JS, the formation of low pressure systems marks the developing stage of CSEs. Whilst the continued formation of the low pressure systems intensifies AL, leading to well established PD resulting in CSE onset. The weakening of jet streak wind speed marks the decaying stage of CSEs due to no PD establishment. To support the development of appropriate diagnostics for detecting the onset of CSEs, a generalized description of the key mechanisms that cause CSE in East Asia is shown schematically in Fig. 5a,b. First, this is based on the analysis of 10 such CSEs (Table 1) in the context of the mechanisms of the different stages leading to CSE onset presented in Supplementary Fig. 2, then depictions of intensification of SH and AL systems and the corresponding PD values response during the surge months (Dec, Jan, Feb) for the reported CSE cases are presented in the Supplementary Fig. 3. Thus, the causative starting point for the development of CSE is the outbreak of cold air masses and strengthening of the PD and the end-point for intensification of CSE is the increase of AL MSLP values and weakening of the PD for CSE onset.

**Table 1:** CSEs classification based on the outbreak of strong cold air at SH (Class I; values in parenthesis indicate monthly averaged SH MSLP value in hPa), the establishment of intense PD (Class II; values in parenthesis indicate the monthly averaged PD value in hPa), and duration (Class III) as resulting from the shift of central position of the jet streak and speed from monthly averaged values.

Rank of CSEs	Class I CSEs Strong cold air outbreak at SH (MSLP in hPa)	Class II CSEs Intense establishment of PD (hPa)	Class III CSEs Jet streak shift, speed change (m/s), AL intensification (hPa) with positive values indicating lowered MSLP, and CSE duration (days)
1	Jan 2011 (1040.3)	Jan 2016 (33.2)	Jan 2008: JS [southwest (3.50° W, 1.5° S), (− 2 m/s)] ⟹ AL (2.47)
			Long duration (21 days)
2	Jan 2016 (1037.3)	Jan 1981 (32.4)	Feb 2008: JS [northeast (7 0° E, 40° N), (− 6 m/s)] ⟹ AL (0.94)
			Long duration (16 days)
3	Dec 2005 (1037.1)	Dec 2005 (31.3)	Jan 2016: JS [northeast (12.5° E, 1.5° N), (1 m/s)] ⟹ AL (− 5.73)
			Average duration (8 days)
4	Jan 2008 (1036.8)	Jan 2011 (30.6)	Feb 2012: JS [northeast (3° E, 4° N), (6 m/s)] ⟹ AL (− 1.85)
			Short duration (7 days)
5	Jan 1981 (1033.9)	Jan 2008 (24.5)	Jan 1981: JS [northeast (12.5° E, 1.5° N), (11 m/s)] ⟹ AL(− 8.33)
			Short duration (6 days)
6	Dec 2001 (1036.2)	Dec 2001 (26.5)	Dec 2009: JS [eastern (5° E, no change), (− 2 m/s)] ⟹ AL (− 0.75)
			Short duration (6 days)
7	Jan 1984 (1033.2)	Feb 2012 (21.9)-yes	Dec 2001: JS [eastern (5° E, no change), (3 m/s)] ⟹ AL (− 1.13)
			Short duration (6 days)
8	Feb 2012 (1031.6)	Jan 1984 (21.7)	Jan 1984: JS [southeast (7.5° E, 1.5°S), (8 m/s)] ⟹ AL (1.67)
			Short duration (5 days)
9	Feb 2008 (1031.3)	Feb 2008 (20.5)	Jan 2011: JS [ southeast (2.5° E, 1.5° S), (12 m/s)]⟹ AL (− 4.23)
			Short duration (5 days)
10	Dec 2009 (1029.5)	Dec 2009 (19.3)	Dec 2005: JS [southeast (7° E, 1°S), (16 m/s)] ⟹ AL (− 5.15)
			Short duration (5 days)
NA	Jan 2000 (1034)	Jan 2000 (19.73)-no	Jan 2000: JS [northwest (18° W, 0.5° N), (− 12 m/s)] ⟹ AL (5.17)
NA	Jan 2006 (1031)	Jan 2006 (21.0)	Jan 2006: JS [no shift (no change, no change), (− 2 m/s)] ⟹ AL (1.17)
NA	Jan 1997 (1028)-no	Jan 1997 (19.72)-no	Jan 1997: JS [northeast (12.5° E,1° N), (2 m/s)] ⟹ AL (− 1.6)
NA	Jan 2014 (1027)-no	Jan 2014 (19.65)-no	Jan 2014: JS [northeast (10° E, 2° N), (0 m/s)] ⟹ AL (− 1.82)

The four cases with rank as “NA” denote CSE in Class I that failed to give rise to Class II, or from Class II to Class III. Reported CSEs information is obtained from Emergency Events Database EM-Dat. (–) values represent the value below the climatological mean.

**Figure 6:**
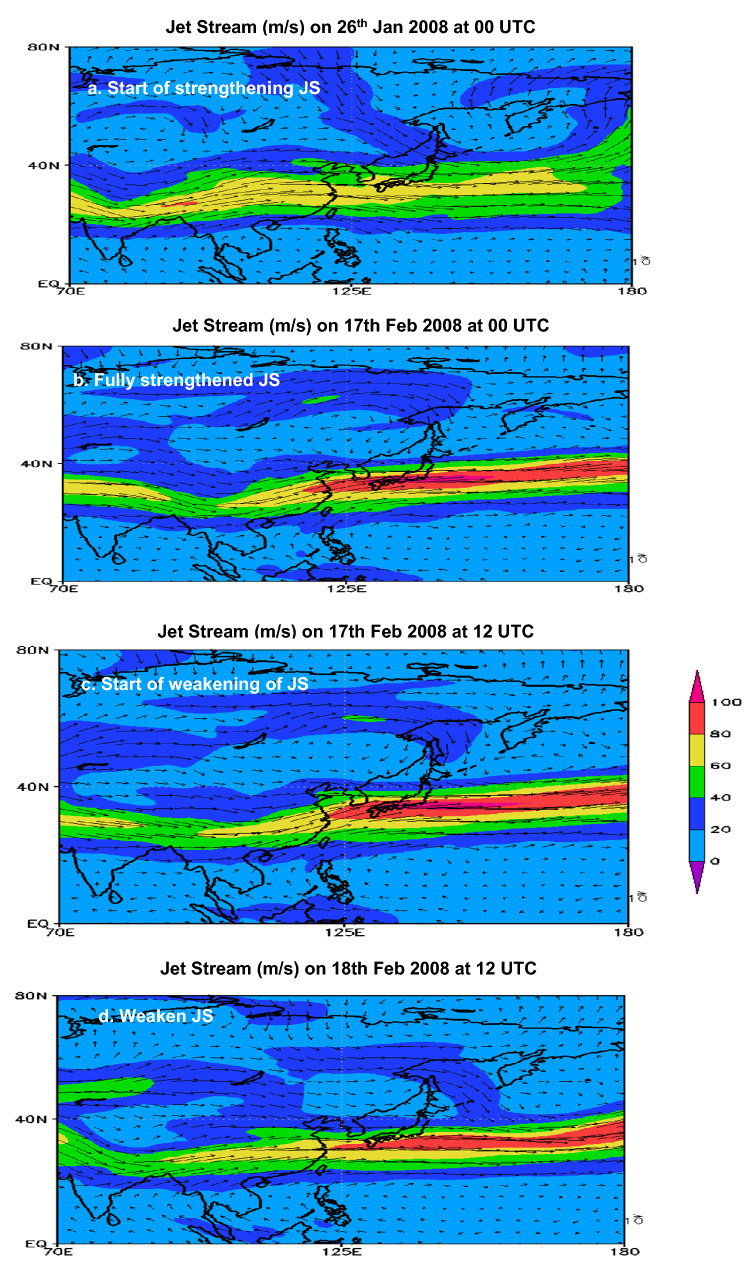
Spatial distribution of strengthening, fully strengthened and weakened JS progressing eastward during CSE over Jan–Feb 2008. (**a**) Start of strengthening of the JS (**a**) on 26th Jan 2008 at 00 UTC; (**b**) Fully strengthened JS with jet streak wind speed reaching above 100 m/s (red color); (**c**) start of weakening of JS marked by the split in JS; (**d**) Weaken JS with jet streak wind speed dropping below 100 m/s (orange color).

**Figure 7:**
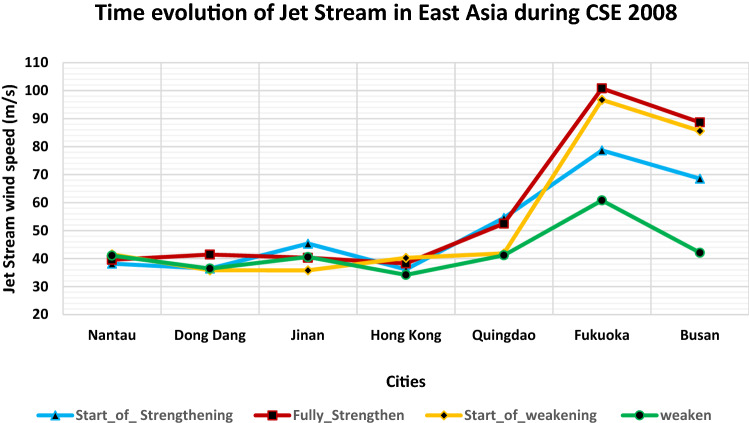
Time evolution of Jet Stream in East Asia during CSE 2008. (**a**) Start of strengthening of the JS (**a**) on 26th Jan 2008 at 00 UTC; (**b**) fully strengthened JS with jet streak wind speed reaching 100 m/s; (**c**) start of weakening of JS marked by the split in JS; (**d**) weaken JS marked with low wind speed.

We have seen that jet streak plays a key role in the formation of the low pressure system. Apart from the intensification of the jet streak, the pattern of JS also significantly influences the progression of CSEs in terms of their stability. Jet streak strength is also dependent on its longitudinal extent in manifesting the role of diverging air masses in inhibiting the intensification of CSEs at the surface. For example, the larger longitudinal extent of jet streak relative to its climatology leads to a fully strengthen jet streak resulting in the formation and development of intense low pressure systems. For example, under the influence of a fully developed jet streak with longitudinal extent > 15°, intense low pressure systems resulted in the strongest AL anomaly (supplementary Table 1) leading to strong CSEs during Jan 2016, Dec 2005, Jan 1981. Whereas, the smaller extent of jet streak relative to its climatology leads to a comparatively less intense jet streak and thus lesser intense AL as evidenced during CSEs Feb 2012, Jan 2011, Dec 2001, Jan 1984, and Jan–Feb 2008 (Supplementary Table 1). We, therefore, categorize the Jet Stream into two subcategories, one with a fully developed Jet Streak i.e. spanning over 15° in longitude, and the other with a partially developed jet streak i.e. spanning less than 15° along the longitude. Moreover, unusual long lasting CSEs are observed to be associated with partially developed jet streak embedded within a slower progressing jet stream relative to the climate normal. For instance, during the CSE progression over Jan–Feb 2008, a distinct pattern of zonal JS over three different stages is observed that remained stable for a period lasting 20 days. First, the embedded jet streak reaches a stall-like situation, slowing to 50 m/s, well below the climatological mean of 70 m/s. For comparison, the WMO’s adopted minimum JS speed is 30 m/s^27^. The jet streak then starts to strengthen gradually from 60 m/s on 26th Jan 00 UTC (Fig. 6a) and continually increases till 17th Feb 00 UTC reaching a maximum of 100 m/s (Fig. 6b). Second, this pattern of JS has an elongated continuous stretch extending from 70° E up to 180° E. This distinctive JS pattern with an embedded jet streak (within longitude 130° E to 180° E; Fig. 6b), extended beyond its climatological longitudinal extent of 125° E to 165° E (Fig. 2a). Lastly, the JS later started to split from 17th Feb at 12 UTC (Fig. 6c) and further weakened as it moved eastward (Fig. 6d). Over this duration, the regions of Vietnam, South China, Hong Kong, and Taiwan were under the influence of consistent slow moving JS with its speed ranging from 35 to 55 m/s. The slow progression of JS shows no significant difference in wind speeds at the different stages of JS over these regions. Under the continued influence of JS, Hong Kong Observatory recorded the minimum temperatures below 12 °C for 24 consecutive days, making it the longest cold spell in Hong Kong since 1968^28^. In contrast, the regions of South Korea (Busan), and Japan (Fukuoka) experiences an increased wind speed ranging from 40 to 100 m/s due to the influence of jet streak embedded within the JS and also explicitly shows the changes in wind speed at the four different stages of JS (Fig. 7). However, it’s still unclear at this stage what determines the nature of JS patterns. It has been linked that changes in the Arctic are intensifying SH gradually, influencing synoptic pattern^29^. Therefore, one possibility could be the Arctic amplification influencing the upper air and JS patterns, but this requires further investigation.

## Climatological and intraday signatures of CSEs onset

**Figure 8:**
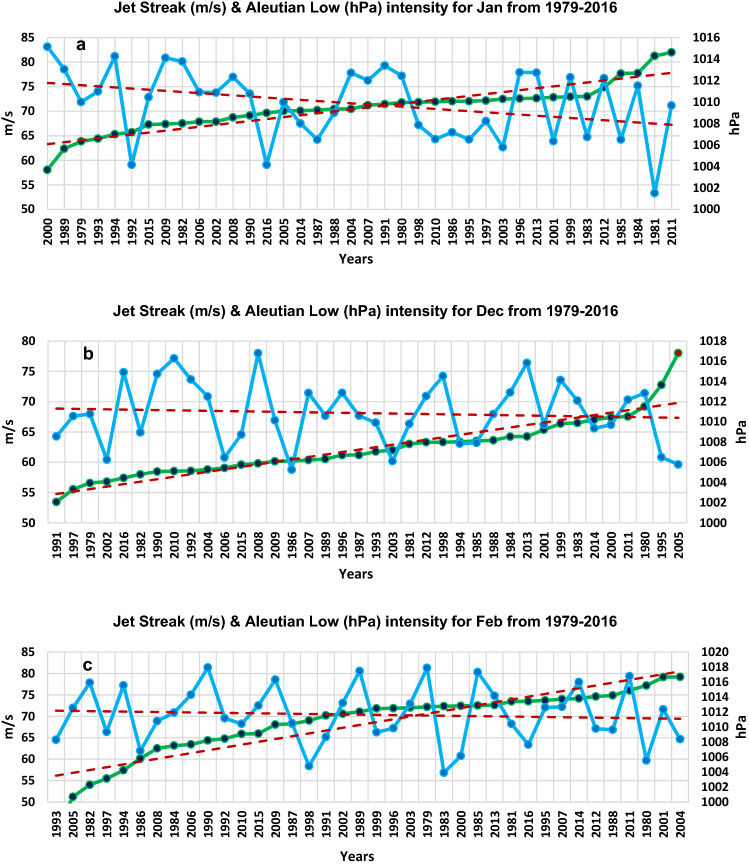
Trend lines for jet streak intensity and MSLP over AL for the month of Jan, Dec, and Feb from 1979–2016. Average of jet streak wind speed (m/s) at 250 hPa (marked with green color) and the average of MSLP (hPa) over AL (marked with blue color) for (**a**) Jan, (**b**) Dec, and (**c**) Feb. Dotted lines in red in (**a**), (**b**) and (**c**) indicates the strengthening years of JS and the corresponding AL intensification years with anti-correlation of (−) 0.414, (−) 0.167 and (−) 0.045 for (**a**), (**b**) and (**c**) respectively. Data from the ECMWF, ERA-Interim.

Figure 8a shows the years of Jan (monthly mean spatially averaged) jet streak wind speed, starting from the lowest in 2000 (left) to the highest in 2011 (right) for a period of 38 years (1979–2016). The corresponding Jan AL fluctuations are also shown. Figure 8b,c show similar plots for Dec and Feb. For Jan, the jet streak was the weakest with an average wind speed of 58 m/s during 2000 while being strongest in 2011 with an average wind speed of 82 m/s. A trend of AL intensification (i.e. lower MSLP values) with the strengthening jet streak wind speed is clearly evident. In particular, the AL minimum of 1001 hPa occurred in 1981 which corresponded to the second largest jet streak speed. Jet streak and AL maintained an anti-correlation of (−) 0.414 for Jan, (−) 0.167 for Dec and (−) 0.045 for Feb (Fig. 5). For the combined Dec–Feb period, the correlation was (−) 0.176.

**Figure 9:**
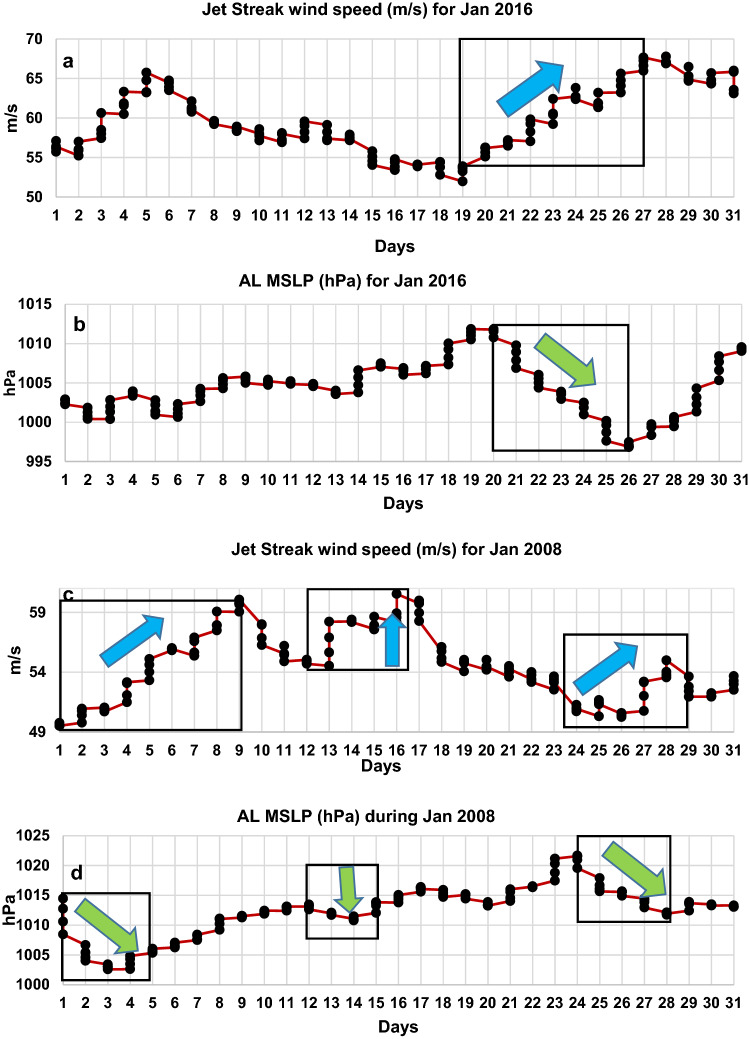
Connection between the strengthening of jet streak wind speed and intensification of AL MSLP during single and multiple episodes CSE onset. (**a**), (**c**) Jet streak wind (m/s) and (**b**), (**d**) corresponding MSLP (hPa) over AL from 1st to 31st Jan of 2016 and 2018, respectively. The blue arrows within boxes in (**a**), (**c**), indicate the strengthening of jet streak while the green arrows within boxes in (**b**), (**d**) indicate the intensification of AL.

The relationship between jet streak and AL is even evident when observed at daily and at intraday (6 hourly) intervals during a strong CSE onset. This relationship also explains the occurrences of a single episode and multiple episodes CSE onset. For example, just before the onset of the most intense week-long CSE in Jan 2016 (as based on its PD, Fig. 1b), the jet streak intensity was observed to increase rapidly and persistently for 8 consecutive days over 19th to 27th Jan (Fig. 9a, black box). The jet streak wind speed attained a maximum value of 68 m/s (corresponding to the maximum value attained during that month) on 27th Jan 18 UTC, starting from a value of 52 m/s on 19th Jan 00 UTC. During the same 8 day period, the AL continually dipped from 1011 hPa on 20th Jan 06 UTC to a minimum value of 996 hPa on 26th Jan 06 UTC (corresponding to the minimum value attained during the month) (Fig. 9b, black box). The strength of the jet streak contributes to AL intensification. It is also observed that AL intensifies significantly as a function of jet streak duration, such that the longer duration exhibits higher AL intensification. For example, as the jet streak peaks for 8 days, from 19 to 27th Jan 2016, a consistent intensification in AL MSLP is observed. In contrast, as the jet streak peaks for 5 days from 1st to 5th Jan 2016, the AL MSLP dips initially from 1 to 3 days but then rises for one day and then again dips from 4 to 5th day. Comparatively, with a shorter duration of strengthening the jet streak, an inconsistent drop in AL MSLP is observed. The longer duration of the jet streak allows a continuous progression of diverging air masses at upper air that leads to the formation of intense and frequent low-pressure systems at the surface, resulting in enhanced AL intensification (Fig. 3e). Therefore, under the influence of the diverging air masses of the strengthened jet streak, the continual intensification of AL along with corresponding increased PD led to a severe weather situation that lasted a week over East Asia. Furthermore, AL MSLP becomes more variable as the embedded jet streak strengthens and weakens within the jet stream. For example, for the most stable (long-lived) CSE over Jan-Feb 2008, the jet streak increased rapidly for 7 consecutive days over 1st Jan–9th Jan UTC (Fig. 9c, black box). The jet streak wind speed attained a maximum value of 60 m/s on 9th Jan 06 UTC starting from 49 m/s on 1st Jan 18 UTC. During the same period, a sudden and drastic drop of MSLP over the AL was observed. The MSLP suddenly dipped 12 hPa from 1014 hPa on 1st Jan at 00 UTC to 1002 hPa on 3rd Jan 18 UTC (Fig. 9d, black box). Thereafter it started to rise gradually till 9th Jan at 18th UTC but remained below 1012 hPa i.e. below the climatological normal 1013 hPa pressure. However, when jet streak weakens from 9 to 13th Jan and 17th to 25th Jan, AL MSLP shows only a modest response that seems unlikely to further increase its intensity and remains concentrated primarily with jet streak strength. As seen, three repeated episodes of jet streak strengthening coupled with AL intensification illustrate the casual effect amongst the two that resulted in the longest ever reported CSE in January 2008 over East Asia.

**Figure 10:**
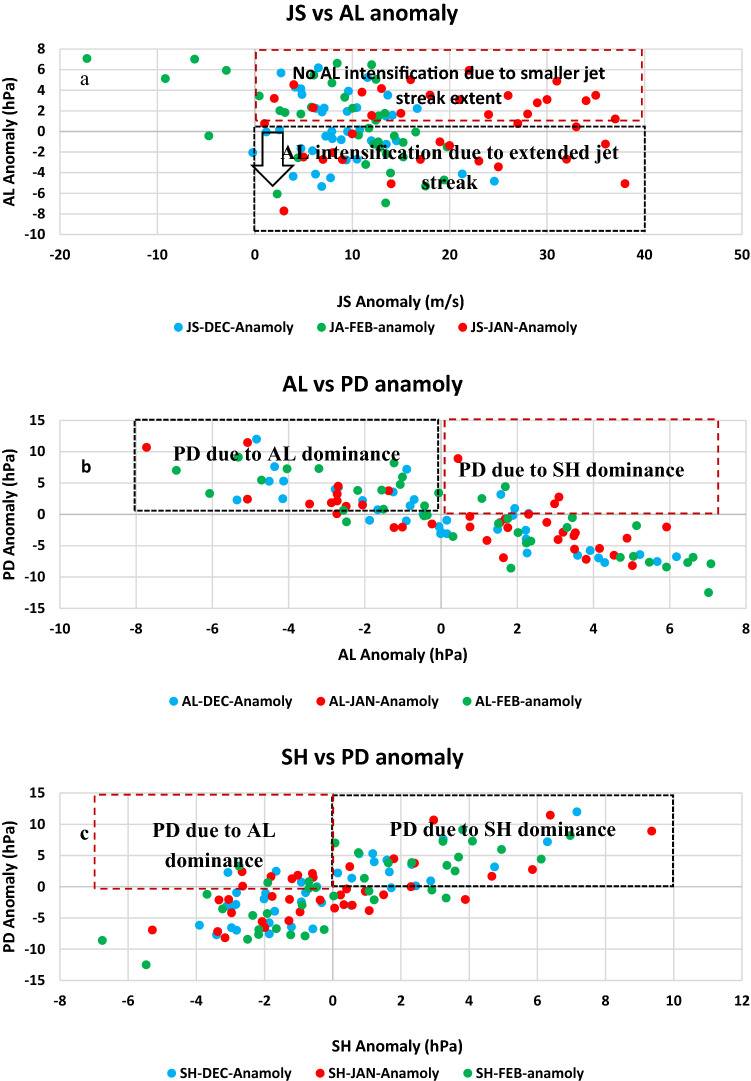
CSEs occurrences based on intensity of jet streak wind speed. Scatter plot of JS anomaly verses AL anomaly (**a**), AL anomaly verses PD anomaly, and SH anomaly verses PD anomaly (**c**) relative to 1981–2010 climate normal period. Correlation coefficient is computed for Dec, Jan, and Feb between (**a**) JS and AL for Dec ((−) 0.1878, p value = 0.2589), Jan ((−)0.4247, p value = 0.007868), and Feb ((−)0.5488, p value = 0.0003596); (**a**,**b**) AL and PD for Dec ((−)0.8411, p value = 3.812e−11), Jan ((−)0.7442, p value = 8.517e−8), and Feb ((−)0.8465 p value = 2.156e−11); (**a**,**b**) SH and PD for Dec ((−)0.7787, p value = 8.557e−9), Jan ((−)0.6907, p value = 0.000001589), and Feb ((−)0.7586, p value = 3.435e−8).

Our further analysis based on long term records (1979–2016) shows that the mechanism driving this response is linked to the embedded jet streak wind speed within the jet stream that influences AL variability. We have seen that the vertical transverse circulations around jet streaks are the significant link that affects the regions within and underneath, leading to the formation of low pressure systems that intensifies AL at the surface (Figs. 2, 3). Scatter plots between JS anomaly and AL anomaly show that that strengthen JS plays a key role in AL intensification (Fig. 10). Strengthen jet streak with a positive anomaly intensifies AL. These cases are shown within the black box in Fig. 10. However, we also observe that strengthened JS with positive anomaly may not intensify AL under certain conditions. There are additional factors that also plays role in intensifying AL apart from jet streak wind speed. This includes pattern of JS in terms of its longitudinal shifts from the climatological mean position. For instance, the westward shift in JS mean position (e.g. Jan 2012 JS anomaly Supplementary Fig. 4a) weakly intensifies AL as the low short lived low pressure systems formed may de dissipated before progressing towards AL. In addition, the presence of a relatively smaller longitudinal spread of JS allows weaker diverging air masses due to lower momentum gained by the air masses within the jet streak and warrants no formation of low pressure systems. Therefore, a Larger westward shift > 3.50 E or the presence of relatively smaller longitudinal spread of JS compared to its climatology (e.g. Jan 2009 JS anomaly Supplementary Fig. 4b) does not intensify AL. These cases are marked with the red box in Fig. 10.

### Proposed CSEs classification

Our results here demonstrate a proximate explanation of CSE onset with the synoptic weather patterns prevailing at that time. We further analyzed 10 strong CSEs that have resulted in severe socio-economic disasters in East Asia as reported in Emergency Events Database (EM-DAT) https://www.cred.be/projects/EM-DAT (Table 1). These CSEs were analyzed and ranked based on three identified classes of synoptic pattern drivers. This comprises the strength of cold air outbreak over SH (Class I), intense progression of CSEs via atmospheric circulations over SH and AL as characterized by PD (Class II), and long lasting CSEs (Class III) as resulting from the directional shifts of JS and its speed from their climatological mean values. Such Class III CSEs have durations extending over a week and up to a month as compared to Class I and Class II CSEs that last for a few days. These climatology means cover over a period of 38 years (1979–2016). For Class I, the monthly mean SH MSLP exceeding 1029 hPa, 1031 hPa, and 1028 hPa for Dec, Jan, and Feb, respectively are taken to trigger cold air outbreaks over SH. For Class II, the establishment of intense SH-AL PD exceeding 18 hPa, 16 hPa, 21 hPa for Dec, Jan, and Feb, respectively either from SH or AL intensification are taken as the criteria for intense progression of CSE. Class III CSEs are ranked by durations and described as long and short durations based on their reported prevailing periods^30–32^. Long duration is those extending over two weeks, while average comprise 1–2 weeks and short less than one week. The duration of the Class III CSEs are further enhanced with the weakening of the jet streak speed.

One advantage of the classification shown in Table 1 is that it allows an inter-comparison of CSEs dominance under the three different synoptic weather drivers. For example, the most severe outbreak of cold air occurred for Jan 2011 CSE under Class I as arising from the strengthening of the SH system above its threshold values. Class II considers an established PD as the SH-AL coupled systems with the strongest PD established during the Jan 2016 CSE. As the PD usually controls the atmospheric circulation and influences the movement of moisture, such Class II CSEs can alter the regional weather situation including a sudden drop in temperature and rise in wind speed. Long-lasting Class III CSEs could be explained by considering both the lower and upper troposphere as a system, specifically the deviation of the central jet streak position and jet streak speed from the climatological mean. This significantly influences the AL intensification arising from the formation of intense low pressure systems. This approach allows an approximate characterization of long lasting CSEs based on jet streak shift, its speed, and AL intensification together influencing Class III CSEs duration as shown in Table 2. In Table 2, the position shift is the primary criteria in identifying the duration of CSEs followed by the jet streak speed as directly correlated with the AL intensification (Fig. 5). Also, the CSE in 2008 extended over two months period severely impacting two regions comprising central China in Jan 2008^2^, and regions of southern China and Hong Kong in Feb 2008^24^. Hence this is considered as two CSEs in EM-DAT and in Table 1. Going down the Class III CSEs in Table 1, the longest duration Class III CSE in Jan 2008 has a westward shift (southwest shift) of the jet streak from its climatological mean and the smallest Jan jet streak speed along with slower eastward progression (Tables 1 and 2, Supplementary Fig. 5a,b). Such a slower eastward progression is via weaker jet streak velocities being lower than the climatological mean of 62 m/s, 70 m/s, and 68 m/s for Dec, Jan, and Feb, respectively. Most importantly, the westward shift (southwest shift) of a slower moving jet streak allowed an abundance of cold air from the polar region to intrude at lower latitude. In contrast, the next four Class III CSE (rank 2 to 5) have eastward shift towards north (northeast shift) of the jet streak (Tables 1 and 2) for the CSE in Feb 2008 (a lower jet streak speed case), and for CSEs in Jan 2016, Feb 2012, and Jan 1981 (higher jet streak speed cases, Supplementary Fig. 5a,c). This slower to faster eastward progression in jet streak results in an increasing (lower to higher) AL intensification, and in decreasing (long to short) duration CSE (Tables 1 and 2). This also holds for the eastward shift of jet streak with no latitudinal shifts during CSE in Dec 2009 and Dec 2001 (Table 1, Class III CSE rank 6–7) except that the CSE durations are short. Additionally, the eastward shift towards the south (southeast shift) of jet streak have been found to be associated only with the faster progressing streak speeds and intensifying AL, all having short CSE durations. The above observations show that CSEs duration are primarily dependent on the position and speed of the jet streak. Longer duration CSEs are likely to occur whenever there is a westward shift of the jet streak, indicating obvious slower eastward progression of JS. The duration is particularly enhanced with the slowdown of the jet streak speed, leading to unusually long CSEs as that occurred during CSE Jan 2008 and Feb 2008 (Table 1, Class III rank 1 and 2). Table 1 further shows that the ranking from the strongest to weakest CSEs for each of the three classes. The top-ranked CSE in Class I as associated with the strongest cold air outbreak ranks 4th in Class II CSE and ranks further lower (rank 9th) in Class III CSE. This means Class I CSEs should be considered as the first indicator, Class II CSEs are the likelihood of Class I CSEs to further intensify and progress based on PD establishment, and finally, Class III CSEs that provide more information on the duration of Class II CSEs from the influence of JS progression and positioning. Moreover, it is also noted that not all strong cold air outbreaks (Class I) lead to Class II or III CSEs. Since Class I CSEs occur due to the cold air outbreak over the Siberia region, the progression of the cold air masses is dominant usually up to Mongolia or may extend up to northern China region depending on the intensity of the outbreak. For example, a CSE that was reported in Jan 2000 was confined over the Mongolia region and remained as a Class I CSE (Supplementary Fig. 6a, and listed in Table 1 under NA). This CSE could not further progress as a Class II CSE due to the weakening of PD. In contrast, the CSE reported in Jan 2006 over Japan region intensified from Class I to Class II CSE due to the strengthening of PD, allowing an abundance of cold air to progress towards the Pacific (Supplementary Fig. 6b; Table 1 under NA). However, this CSE failed to further intensify to be Class III due to only weak intensification of AL under the influence of the jet streak.

**Table 2:** Criteria for classifying Class III CSEs duration.

Jet streak position shift	Jet streak wind speed	AL MSLP intensification	CSE duration (days) based on jet streak shift in position and speed, and AL intensification
Westward	Low	Low	Long duration ≥ 2 weeks
Eastward	(Stall) Low ⟶ high	Low ⟶ high	Long ⟶ short duration ≤ week

High and low values indicate the value above and below the climatological mean. Stall refers to jet streak wind speed well below the climatological mean.

**Figure 11:**
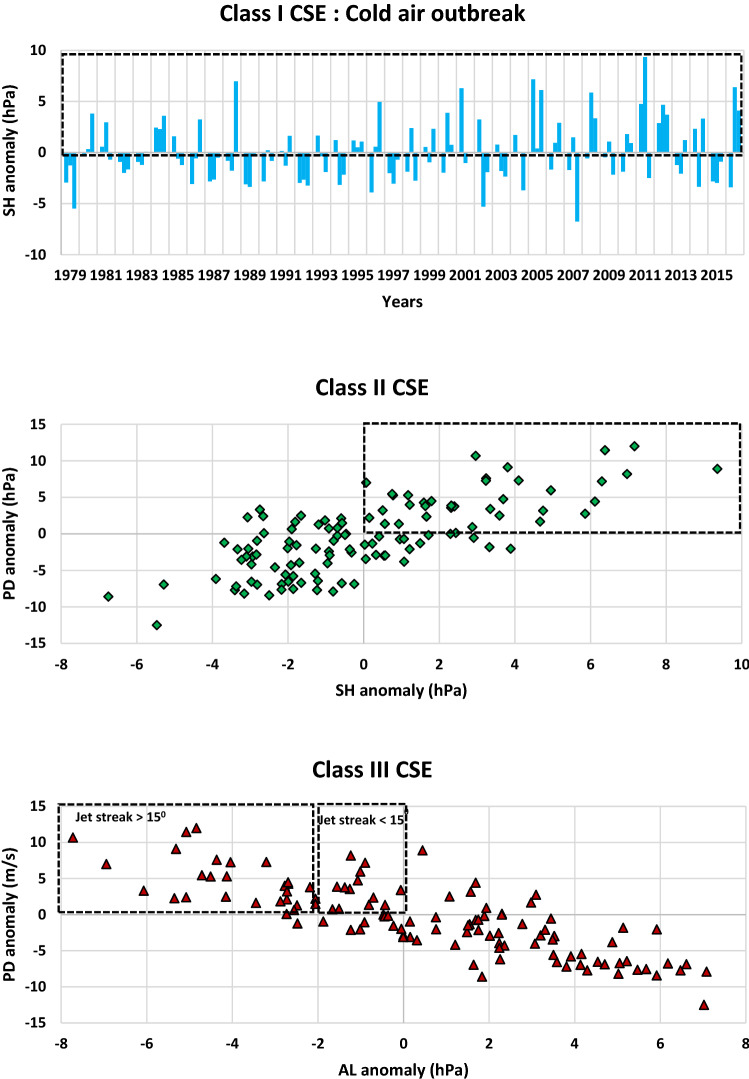
CSEs classification based on SH and AL intensities. Time series plot of cold air outbreaks from 1979–2016 for Dec, Jan, and Feb based on SH anomaly (**a**). Scatter plot of SH anomaly versus PD anomaly (**b**) and AL anomaly versus PD anomaly (**c**) relative to 1981–2010 climate normal period.

These classifications of CSEs are further extended for a long term period (1979–2016) based on the above criteria. All Class I CSEs had an outbreak of cold air masses with SH MSLP anomaly being positive for Dec, Jan, and Feb respectively (Fig. 11). Once CSEs qualify for Class I, the next step is the establishment of the pressure gradient between SH and AL marked with a positive PD anomaly. The PD anomaly then drives the CSEs either as Class II or Class III based on SH or AL dominance respectively. For long lasting Class III CSEs, the mean position of JS is shifted westward within 0–3.5° E from its climatology. Our analysis shows that AL intensification is well correlated with the strengthening of jet streak wind speed for Jan as compared to Dec and Feb. This is likely because the jet streak wind speed climatology reaches its highest value in Jan (63 m/s) followed by Dec (55 m/s) and Feb (60 m/s). Higher jet streak wind speed during Jan results in the formation of more intense low pressure systems intensifying AL. Intense SH marks the outbreak of cold air masses. A strong SH even leads to CSEs for the regions in the vicinity of the outbreak of cold air masses. However, for the progression of these cold air masses at lower latitudes as CSEs, they are dependent on the establishment of PD. AL significantly contributes to the establishment of PD depending on JS wind speed and its longitudinal extent. During the month of Dec, Jan, and Feb, JS progression continually intensifies AL due to the frequent formation of low pressure systems leading to Class II and Class III CSEs, whereas the outbreak of strong cold air masses primarily leads to Class I CSEs alone.

## The way forward

The recent rise in the intensity of SH would make the strong cold air outbreak even stronger (e.g. the Jan 2011, Class I CSE in Table 1) and the larger amplification in PD would probably further intensify CSEs (e.g. Jan 2016, Class-II CSE in Table1). Whilst intensification of AL under the direct influence from JS would lead to more stable long lasting surges (e.g. Jan 2008, Class III CSE in Table 1)). In the recent past, the use of temperature related indicators for interpreting strong CSEs have led to major forecast failures (e.g. 2008, 2016). Therefore, exploring the underlying physics that leads to the onset of CSEs is not only of scientific interest but also of particular use towards reliable predictions.

**Figure 12:**
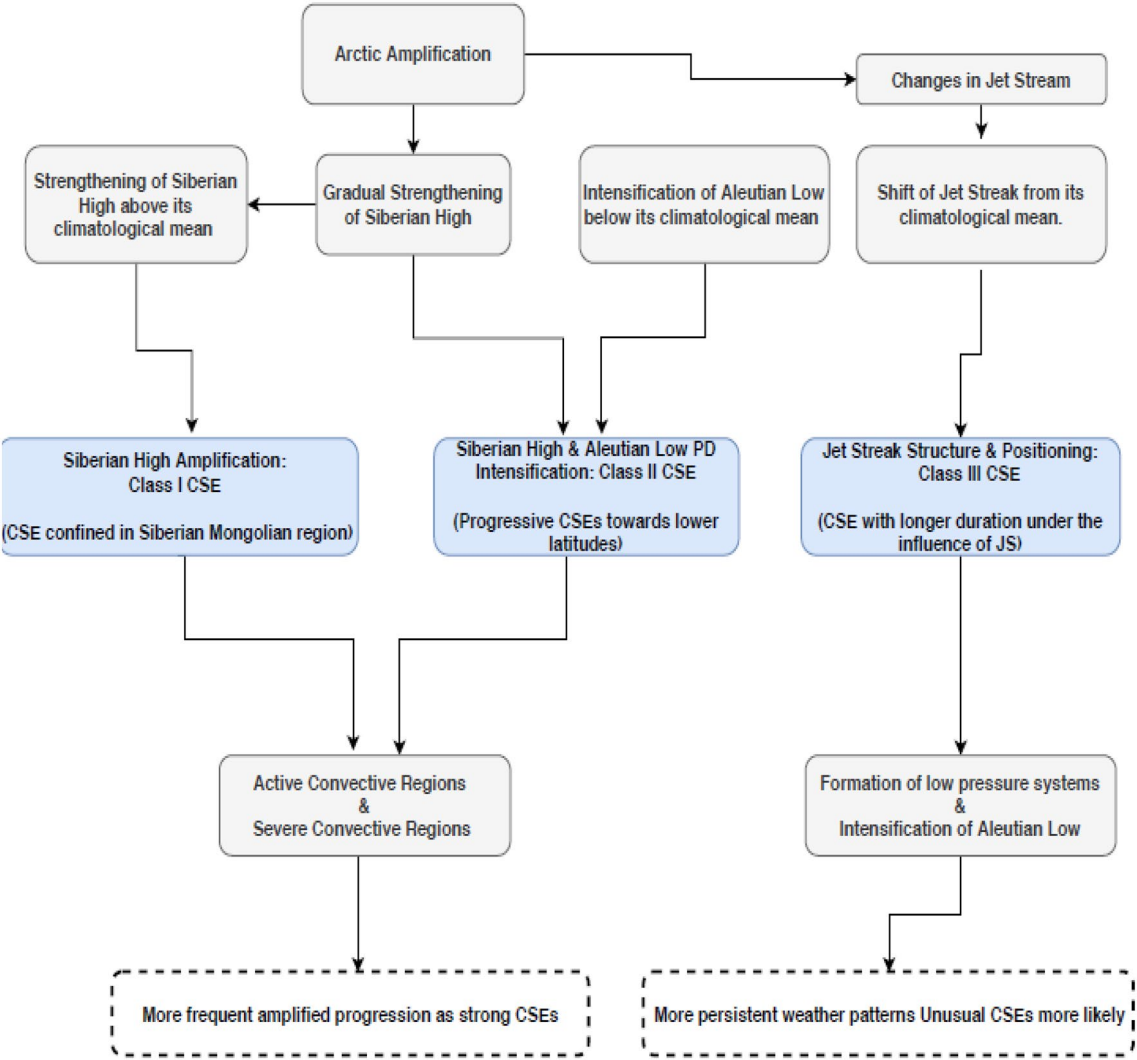
Schematic of ways of CSE occurrence and classification. Pathways that summarize potential mechanisms that contribute to amplified, intensified, and more persistent weather patterns in East Asia leading to CSE onset. The pathway highlighted by blue shaded boxes is presented in the manuscript.

The role of atmospheric circulation has strong implications on how it influences CSE onset. Our analysis shows that the tropospheric disturbances associated with the progression of JS and its longitudinal extent of the embedded jet streak, result in the genesis of the synoptic disturbances at lower troposphere as evident by the formation of low pressure systems. These together lead to strong CSEs onset in East Asia^17^. Moreover, JS positioning from its climatological mean further play a key role in the onset of long lasting CSEs. For example, the unusual extent of JS as extending over southwest China to northern Pacific during the Jan 2008 CSE (Fig. 6) indicated significant progression of the cold air masses at the upper atmosphere. This unusual extent lasted for 14 days continuously leading to colder than average weather over south central China during the period. A schematic showing the pathways that influence the East Asia weather system and CSE onset during the north east monsoon is provided in Fig. 12. Arctic amplification strengthens SH system^17,22^ and alters JS in the high and mid latitudes^33^. The three major systems i.e. SH amplification, coupled SH-AL intensification via the PD, and the position and structure of the jet streak within the JS alter the progression of CSEs. Together these cause changes in CSEs intensity and its propagation direction under the three classes of CSEs.

This research thus shows a new relationship between JS and AL for identifying and characterizing atmospheric process that leads to CSEs classification in East Asia. The classification allows an inter-comparison of CSEs dominance and provides insight into the mechanism leading to unusual CSEs**.** Besides requiring a fuller understanding of this relationship, possible further steps are to investigate and characterize CSEs based on possible contributions of from the mid-latitude or high-latitude processes such as the impact of Arctic amplification on temperature extremes to further enhance deciphering of casual linkages that enable CSEs predictions for impact and risk based forecasting.

## Methods

Dataset (year 1979–2016) obtained from the European Centre for Medium Range Weather Forecasts (ECWMF) (https://www.ecmwf.int/en/forecasts/datasets/reanalysis-datasets/era-interim). Analysis was performed using the analysis fields from ERA-Interim global data archived at ECMWF (archival since 1979). The dataset was post processed to use a 4-dimensional data environment using the Grid Analysis and Display System (GrADS) software (http://www.dmc.fmph.uniba.sk/public_html/doc/grads/head.html). Standard statistical analysis was performed on the data sets using Mann–Kendall (MK) test to determine the monotonic decreasing trend on the time series of the meteorological variables. For measuring the statistical relationship between AL and JS system, Pearson’s correlation coefficient was computed based on the method of covariance.

## Supplementary Information

Supplementary Information.

## References

[1] Chen TC, Yen MC, Huang WR, Gallus WA (2002). An East Asian cold surge: Case study. Mon. Weather Rev..

[2] Zhou B, Gu L, Ding Y, Shao L, Wu Z, Yang X, Li C, Li Z, Wang X, Cao Y, Zeng B, Yu M, Wang M, Wang S, Sun H, Duan A, An Y, Wang X, Kong W (2011). The Great 2008 Chinese Ice Storm: Its socioeconomic-ecological impact and sustainability lessons learned. Bull. Am. Meteorol. Soc..

[3] Chan JCL, Li CY, Chang C-P (2004). The East Asia winter monsoon. East Asian Monsoon, World Scientific Series on Asia-Pacific Weather and Climate.

[4] Jeong JH, Kim BM, Ho CH, Noh YH (2008). Systematic variation in wintertime precipitation in East Asia by MJO-induced extratropical vertical motion. J. Clim..

[5] Chang CP (2011). The Global Monsoon System: Research and Forecast.

[6] Tangang, F. T. *et al.* On the roles of the northeast cold surge, the Borneo vortex, the Madden-Julian Oscillation, and the Indian Ocean Dipole during the extreme 2006/2007 flood in southern peninsular Malaysia (2008).

[7] Wu MC, Chan JCL (1995). Surface features of winter monsoon surges over South China. Mon. Weather Rev..

[8] Yang T, Wu P, Vivian YC, Huey S (2009). Cold surge: A sudden and spatially varying threat to health. Sci. Total Environ..

[9] Wang XY, Wang K, Su L (2016). Contribution of atmospheric diffusion conditions to the recent improvement in air quality in China. Sci. Rep..

[10] Takahashi HG, Fukutomi Y, Matsumoto J (2011). The impact of long-lasting northerly surges of the East Asian winter monsoon on tropical cyclogenesis and its seasonal March. J. Meteorol. Soc. Jpn.

[11] Park TW, Ho C-H, Jeong S-J, Choi Y-S, Park SK, Song C-K (2011). Different characteristics of cold day and cold surge frequency over East Asia in a global warming situation. J. Geophys. Res..

[12] Gong DY, Ho CH (2004). Intra-seasonal variability of wintertime temperature over East Asia. Int. J. Climatol..

[13] Takaya K, Nakamura H (2005). Mechanisms of intraseasonal amplification of the cold Siberian high. J. Atmos. Sci..

[14] Bell GD, Halpert MS, Ropelewski CF, Kousky VE, Douglas AV, Schnell RC, Gelman ME (2000). Climate assessment for 1999. Bull. Am. Meteorol. Soc..

[15] Lawrimore JH, Halpert MS, Bell GD, Menne MJ, Lyon B, Schnell RC, Gleason KL, Easterling DR, Thiaw W, Wrightand WJ, Heim RR (2000). Climate assessment for 2000. Bull. Am. Meteorol. Soc..

[16] Ye SL, Duan WS (2015). Interannual relationship between the winter Aleutian low and rainfall in the following summer in South China. Atmos. Oceanic Sci. Lett..

[17] Kumar A, Lo EY, Switzer AD (2019). Relationship between East Asian cold surges and synoptic patterns: A new coupling framework. Climate.

[18] Ding Y, Krishnamurti TN (1987). Heat budget of the Siberian high and the winter monsoon. Mon. Weather. Rev..

[19] Jeong J-H, Ho C-H (2005). Changes in occurrence of cold surges over East Asia in associated with Arctic Oscillation. Geophys. Res. Lett..

[20] Li QP, Ding YH, Dong WJ, Yang GH (2007). A numerical study on the winter monsoon and cold surge over East Asia. Adv. Atmos. Sci..

[21] Li Q, Yang S, Wu T, Liu X (2017). Sub-seasonal dynamical prediction of East Asian cold surges. Weather. Forecast..

[22] Zhao S, Feng T, Tie X, Long X, Li G, Cao J, Zhou W, An Z (2018). Impact of climate change on Siberian high and wintertime air pollution in china in past two decades. Earth’s Future.

[23] Pithan F, Mauritsen T (2014). Arctic amplification dominated by temperature feedbacks in contemporary climate models. Nat. Geosci..

[24] Wagner J (1981). Weather and circulation of January 1981. Mon. Weather Rev..

[25] WMO. WMO statement on the state of the global climate in 2017; WMO-No-1212. ISBN 978-92-63-11212-5 (WMO, 2017).

[26] WMO. WMO Statement on the State of the Global Climate in 2019. https://public.wmo.int/en/media/press-release/2019-concludes-decade-of-exceptional-global-heat-and-high-impact-weather (2020).

[27] WMO. 1958. Observational Characteristics of the Jet Stream. https://library.wmo.int/doc_num.php?explnum_id=1725

[28] HKO (Hong Kong Observatory). Very cold weather afflicted Hong Kong. http://www.hko.gov.hk/press/SP/pre20160124.htm (2016).

[29] Zhao S, Feng T, Tie X, Long X, Li G, Cao J, Zhou W, An Z (2018). Impact of climate change on Siberian high and wintertime air pollution in China in past two decades. Earths Future.

[30] CRED. Emergency Events Database. https://www.cred.be/projects/EM-DAT (2020).

[31] Ding Y, Krishnamurti T (1987). Heat budget of the Siberian high and the winter monsoon. Mon. Weather Rev..

[32] Park TH, Ho CH, Yang S, Jeong JH (2010). Influences of Arctic Oscillation and Madden-Julian Oscillation on cold surges and heavy snowfalls over Korea: A case study for the winter of 2009–2010. J. Geophys. Res..

[33] Cohen J, Screen JA, Furtado JC, Barlow M, Whittleston D, Coumou D, Francis J, Dethloff K, Entekhabi D, Overland J, Jones J (2014). Recent Arctic amplification and extreme mid-latitude weather. Nat. Geosci..

