# Supplementary material for: A new approach to cold surge classification in East Asia
Source: Sci Rep. 2021 Dec 8;11:23659. doi: 10.1038/s41598-021-02873-0 (PMC8654882; doi:10.1038/s41598-021-02873-0)
Supplement: Supplementary file 1 — Supplementary Information. [file 41598_2021_2873_MOESM1_ESM.docx]

Article

A new approach to cold surge classification in East Asia

Anupam Kumar ^1,2*^

^1^ Institute of Catastrophe Risk Management, Nanyang Technological University, Singapore

^2^ Solar Energy Research Institute of Singapore, National University of Singapore, Singapore

***** Correspondence: seranup@nus.edu.sg

Received: date; Accepted: date; Published: date

Outer region

Inner region

**Supplementary Figure 1. Synoptic composites of (a) strengthening of jet streak wind speed and (b) intensification of AL along the longitudes based on 31 days average in Jan 2016. The i**nner and outer boundary of jet streak wind speed is marked as Inner and outer region in (a). As the air parcel enters the jet streak (extreme left) it accelerates and reaches its maximum speed at the centre and finally decelerates as it leaves the jet streak (extreme right). AL MSLP starts decreasing (intensifying) towards the eastern longitude within the LPS domain during Jan 2016 (blue line).

**Supplementary Figure 2.** Anomaly plots for (a) Siberian High (hPa), (b) Aleutian Low (hPa), (c) Pressure Difference (hPa), and Jet Stream (m/s) during reported CSEs.

Supplementary Figure 3: Onset of CSEs in East Asia. Higher MSLP SH values lead to the development of CSE. Lower MSLP AL values intensify CSE. The well-established PD marks the onset of CSEs, whereas a dropping PD weakens CSEs progression.

**
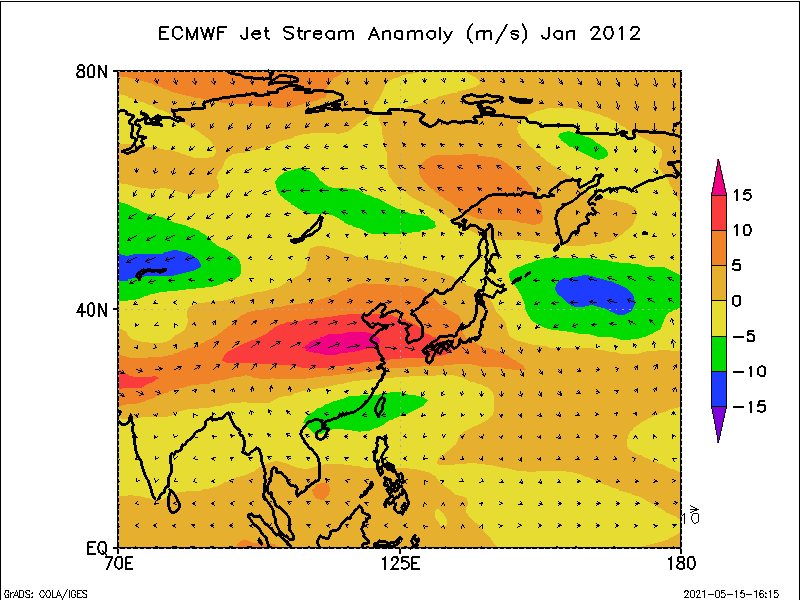
**

ECMWF Jet Stream anomaly (m/s) Jan 2012

**a**

**
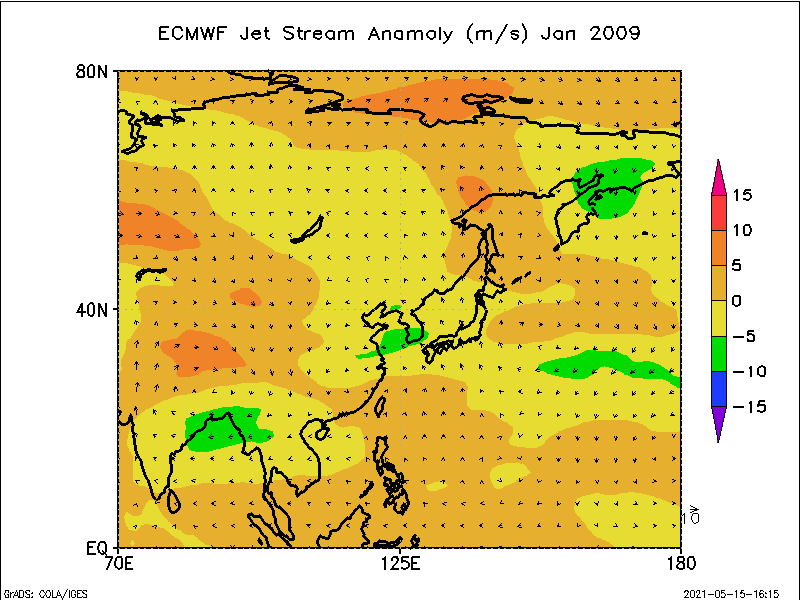
**

ECMWF Jet Stream anomaly (m/s) Jan 2009

**b**

**Supplementary Figure 4.** Anomaly plots for Jet Stream (m/s) during (a) Jan 2012 and (b) Jan 2009.

**Jet Stream (m/s) Jan 1979-2016**


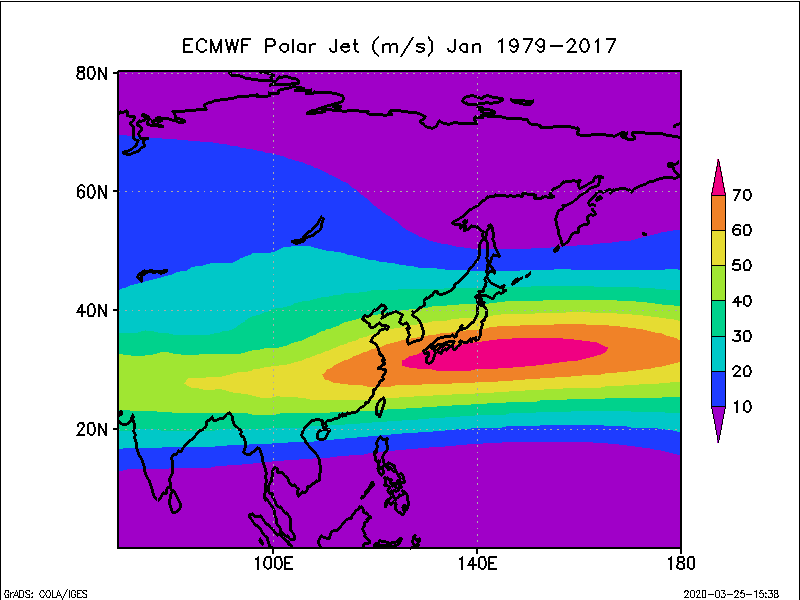


**a.**

**Jet Stream (m/s) Jan 2008**


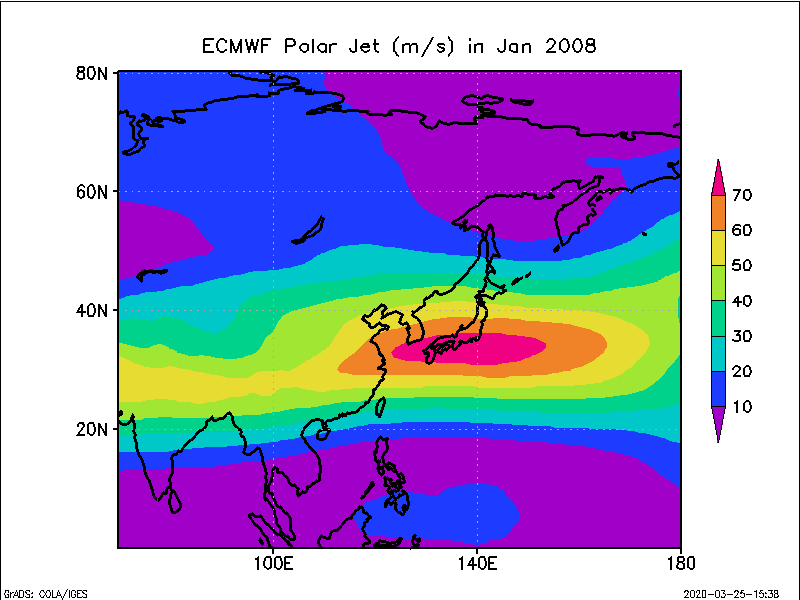


**Jet Stream (m/s) Jan 2016**

**b.**


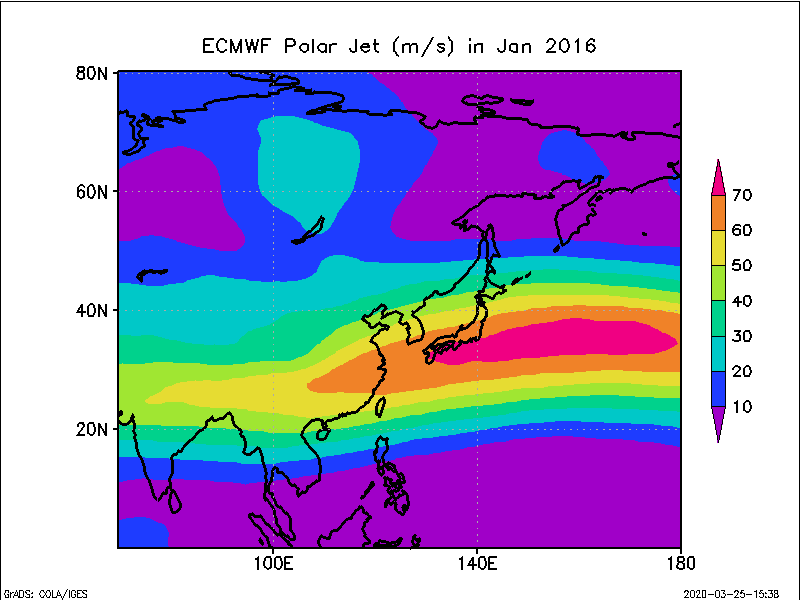


**c.**

**Supplementary Figure 5.** Position of easterly Jet Stream showing shift and speed of embedded jet streak (m/s) during (a) Jan 1979-2016, (b) Jan 2008 (westward shift), and (c) Jan 2016 (eastward shift). X mark in the figures represents the centroid of the jet streak.

**Class I CSE Jan 2000**


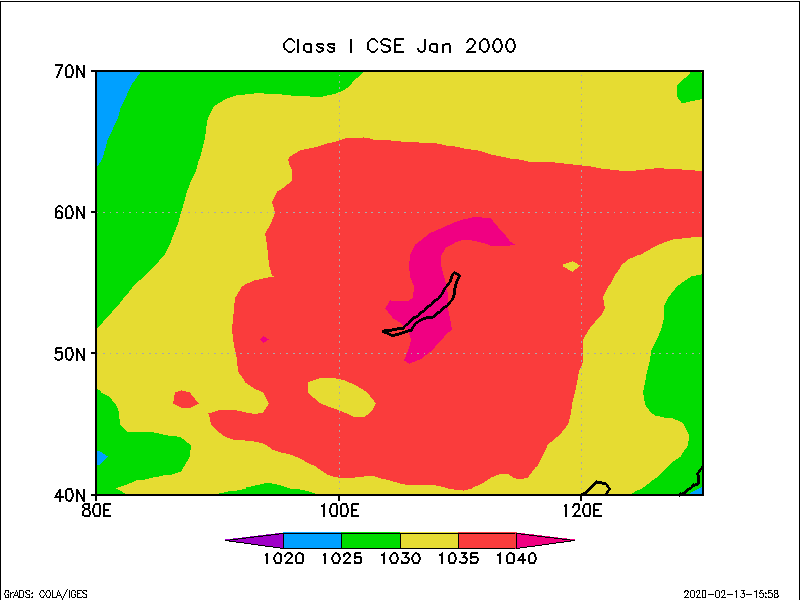


**Class II CSE Jan 2006**

**a.**


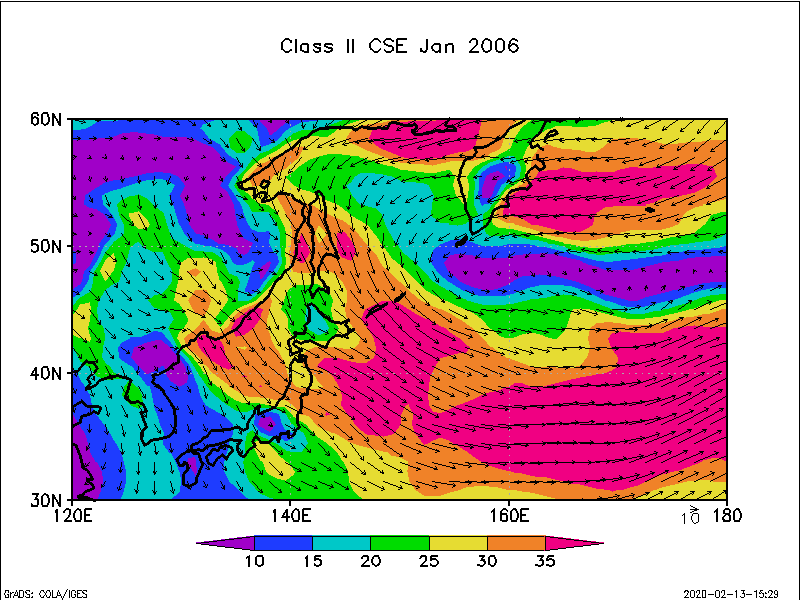


**b.**

**Supplementary Figure 6.** Spatial distribution of (a) Surface pressure for Class I CSE during Jan 2000 and (b) Surface wind fields (km/hr) for Class II CSEs during Jan 2006 progressing towards the Pacific. In (a) Lake Baikal in Siberia are represented by the black thick line.

**Supplementary Table 1: Class III Type CSEs**

| **Year** | **Month** | **SH anomaly** | **AL Anomaly** | **Jet Streak Extent** | **SH-AL PD anomaly** |
| --- | --- | --- | --- | --- | --- |
| 2016 | Jan | 6.38 | -6.14 | >15^0^ | 12.52 |
| 2012 | Feb | 3.69 | -1.94 | <15^0^ | 5.63 |
| 2011 | Jan | 9.35 | -1.45 | <15^0^ | 10.80 |
| 2009 | Dec | -0.30 | -2.08 | <15^0^ | 1.78 |
| 2008 | Jan | 5.85 | 2.67 | <15^0^ | 3.18 |
| 2008 | Feb | 3.34 | -1.47 | <15^0^ | 4.81 |
| 2005 | Dec | 7.16 | -6.23 | >15^0^ | 13.39 |
| 2001 | Dec | 6.29 | -0.97 | <15^0^ | 7.26 |
| 1984 | Jan | 2.29 | 0.74 | <15^0^ | 1.55 |
| 1981 | Jan | 2.96 | -8.89 | >15^0^ | 11.85 |
